# Toward objective brain monitoring of cancer-related cognitive impairment: an extracellular vesicle–based conceptual framework

**DOI:** 10.3389/fonc.2026.1926793

**Published:** 2026-10-20

**Authors:** Masato Mitsuhashi

**Affiliations:** NanoSomiX, Inc., Aliso Viejo, CA, United States

**Keywords:** biomarkers, brain health, cancer-related cognitive impairment, extracellular vesicles, liquid biopsy, precision medicine

## Introduction

Advances in cancer detection and treatment have substantially improved survival across many malignancies, increasing attention to long-term health outcomes and quality of life (QOL) (1, 2). Cancer-related cognitive impairment (CRCI), often referred to colloquially as “chemobrain,” may occur before, during, or after anticancer therapy and can affect multiple cognitive domains, including learning and memory, attention, processing speed, and executive function (3–6). The severity, affected domains, and temporal trajectories vary considerably among individuals, indicating that CRCI is a heterogeneous rather than uniform or sequential process (3, 4, 6).

Current CRCI assessment relies primarily on patient-reported symptoms and standardized neuropsychological testing, which provide complementary but not necessarily concordant information (4, 5, 7). Although essential for characterizing cognitive symptoms and performance, these approaches provide limited information about the underlying biological processes occurring within the brain. The biological basis of CRCI is multifactorial and may involve interacting inflammatory, metabolic, neurovascular, synaptic, and glial alterations resulting from cancer, host responses, individual susceptibility, and anticancer therapies (4, 8–10). These processes are difficult to assess directly and longitudinally in the living human brain, creating a need for minimally invasive approaches that can provide complementary molecular information.

Circulating extracellular vesicle (EV) populations enriched for brain-associated cell markers represent one potential source of such information. Brain cells release EVs that can reach the peripheral circulation, and circulating EV populations associated with specific neural cell types are increasingly being investigated as blood-based sources of brain-related molecular information (11–14). EV populations enriched using neuron-associated markers, commonly referred to as neuron-derived EVs (NDEs) (11–13), and those enriched using oligodendrocyte-associated markers, commonly referred to as oligodendrocyte-derived EVs (ODEs) (14), have been investigated as candidate sources of neuronal and oligodendroglial information, respectively. ODE measurements may provide information related to oligodendrocyte and myelin biology, whereas NDE measurements may provide complementary information regarding neuronal and synaptic processes. These approaches remain investigational, however, and require rigorous analytical and clinical validation, including evaluation of marker specificity, pre-analytical variability, and potential systemic effects of cancer and its treatment on circulating EV profiles.

In this Perspective, we discuss the clinical and biological complexity of CRCI and propose a conceptual framework for integrating brain-associated EV measurements with neuroimaging and cognitive assessments. Rather than proposing EVs as stand-alone diagnostic biomarkers, we consider their potential role as a complementary molecular component of longitudinal brain health monitoring in oncology.

## Clinical importance and unmet need

CRCI can substantially affect daily functioning, employment, medication management, independence, and QOL (2, 3, 7). Its clinical expression is heterogeneous, with variation in affected domains, onset, severity, and persistence across individuals (3, 4, 6), complicating identification of patients at increased risk and longitudinal assessment of cognitive trajectories.

A major unmet need is the absence of an established framework for objective longitudinal assessment of the biological changes associated with CRCI. Unlike other treatment-related toxicities, such as cardiotoxicity, hepatotoxicity, nephrotoxicity, and myelosuppression, which can be monitored using established clinical, laboratory, and imaging-based measures, CRCI lacks comparable tools for routinely assessing underlying brain-related biological changes. Neuropsychological testing and patient-reported outcomes remain essential for characterizing cognitive performance and symptoms but may be constrained by time, expertise, repeated-testing effects, and limited scalability in routine oncology practice (5, 7, 15). Moreover, these approaches do not directly assess the molecular and cellular processes underlying CRCI.

This gap highlights the need for minimally invasive molecular measures that can be obtained repeatedly across the cancer care continuum. Such measures could help define when brain-related biological changes emerge, how they relate to cognitive trajectories, and whether they persist or resolve after therapy. Ultimately, scalable biological measures integrated with cognitive assessment and other complementary approaches could facilitate investigation of individual risk, longitudinal recovery, and potential neuroprotective or rehabilitative interventions (2, 7).

## Biological alterations underlying CRCI

The biological basis of CRCI is multifactorial, reflecting interacting effects of cancer, systemic host responses, individual susceptibility, and anticancer therapies rather than a single neurotoxic pathway. Proposed mechanisms include neuroinflammation, oxidative stress and mitochondrial dysfunction, blood–brain barrier and neurovascular dysfunction, impaired neurogenesis and synaptic plasticity, and oligodendrocyte and white matter injury (8–10, 16) (**Figure 1**). These processes may involve multiple cellular components of the brain, including neurons, oligodendrocytes, astrocytes, microglia, and cells of the neurovascular unit, and may disrupt cellular homeostasis, synaptic connectivity, myelination, and neural networks supporting memory, attention, processing speed, and executive function (8–10, 16). Importantly, such alterations are not restricted to chemotherapy; cancer itself and other therapeutic modalities may contribute through overlapping biological pathways (9, 10). Thus, CRCI may reflect heterogeneous and dynamic changes across multiple brain cellular compartments, providing a rationale for minimally invasive biomarkers capable of capturing underlying molecular processes.

**Figure 1:**
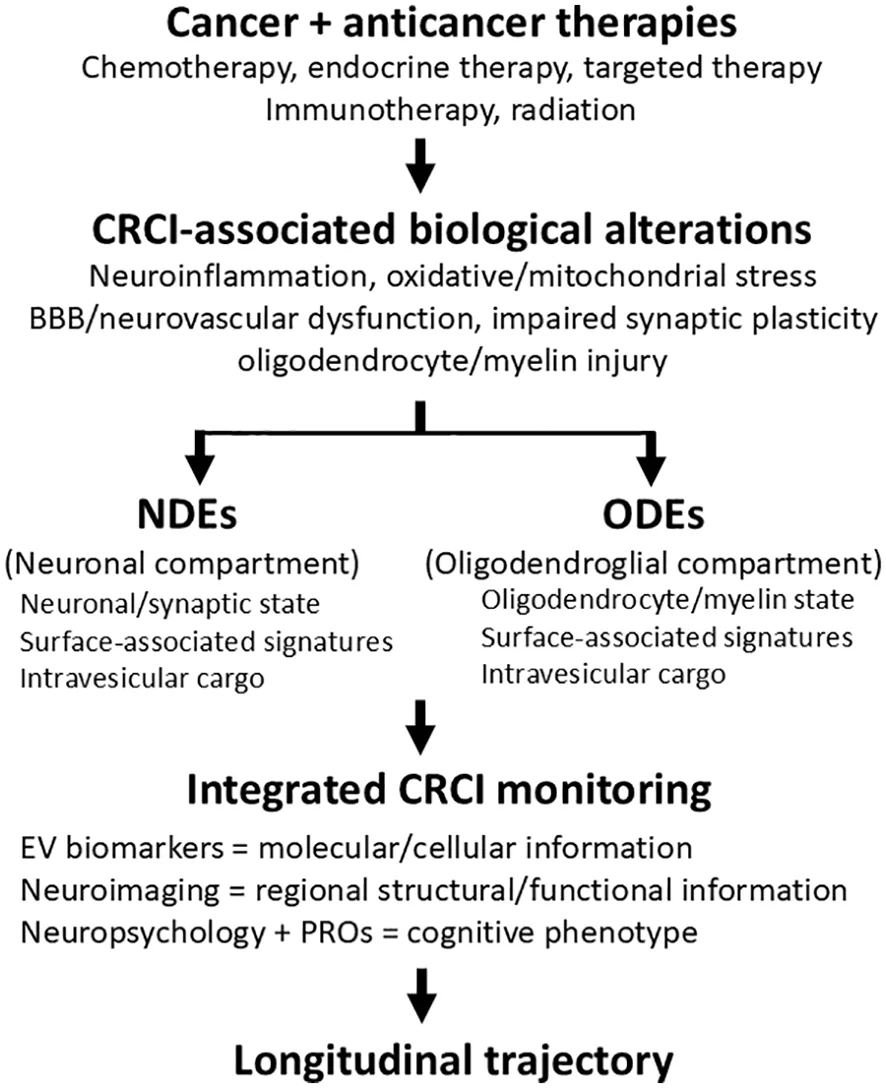
Proposed multimodal framework for extracellular vesicle–based monitoring of cancer-related cognitive impairment (CRCI). Cancer and anticancer therapies may contribute to CRCI through interacting biological processes, including neuroinflammation, oxidative stress and mitochondrial dysfunction, blood–brain barrier and neurovascular alterations, impaired neurogenesis and synaptic plasticity, and oligodendrocyte/myelin injury. These processes may affect multiple brain cellular compartments and neural networks underlying cognitive function. Within the proposed framework, circulating EV populations enriched using neuron-associated markers (NDEs) and oligodendrocyte-associated markers (ODEs) may provide complementary molecular information regarding neuronal/synaptic and oligodendrocyte/myelin-related processes, respectively. EV analysis may incorporate cell-associated markers, surface-associated molecular signatures, and intravesicular cargo, including proteins, nucleic acids, lipids, and metabolites. Because circulating brain-associated EV populations do not currently provide reliable anatomical localization, EV-derived molecular information is envisioned as complementary to neuroimaging, which provides regional structural and functional information, and to neuropsychological assessment and patient-reported outcomes (PROs), which characterize objective cognitive performance and subjective cognitive symptoms, respectively. Integration of these modalities across pretreatment, treatment, and survivorship may enable investigation of longitudinal relationships among molecular alterations, brain structure and function, and cognitive outcomes. This framework does not assume a universal temporal sequence of white- and gray-matter alterations, which may occur concurrently or follow different trajectories among individuals. The framework remains investigational and requires prospective analytical and clinical validation.

Genetic and epigenetic factors may further contribute to interindividual susceptibility to CRCI. Candidate-gene studies have investigated polymorphisms in apolipoprotein E (APOE), catechol-O-methyltransferase (COMT), brain-derived neurotrophic factor (BDNF), and genes involved in DNA repair and oxidative-stress responses, although reported associations with cognitive outcomes have been inconsistent (17). Anticancer therapies may also induce genomic and epigenomic alterations relevant to neural function. In patients with breast cancer, chemotherapy has been associated with changes in peripheral leukocyte DNA methylation, some of which were associated with perceived cognitive impairment (18). Genetic variation in DNA methyltransferase 1 (DNMT1) has also been associated with selected self-reported cognitive outcomes during chemotherapy, although not with objective cognitive impairment (19). Experimental evidence further indicates that cisplatin-induced DNA lesions can trigger DNA repair–dependent double-strand breaks and neuronal death, providing a potential mechanistic link between platinum-associated genotoxic stress and neuronal injury (20). However, direct evidence linking treatment-induced genomic or epigenomic alterations in human brain cells to CRCI remains limited. These findings support further investigation of genetic susceptibility and treatment-associated genomic and epigenomic changes as contributors to CRCI heterogeneity.

## Proposed conceptual framework

Neuroimaging studies indicate that CRCI may involve both white- and gray-matter alterations, including changes in frontal and limbic regions and distributed functional networks (21, 22). CRCI therefore likely reflects heterogeneous involvement of multiple brain compartments rather than injury confined to a single anatomical region, supporting approaches that capture complementary dimensions of cancer- and treatment-associated brain changes.

Based on this model, we propose circulating EV populations enriched for brain-associated cell markers as minimally invasive sources of molecular information potentially related to neural cellular processes. Although astrocytes and microglia contribute importantly to brain homeostasis and CRCI-related inflammatory and metabolic responses, the present framework focuses on neurons and oligodendrocytes because they represent two complementary components of neural network function: neuronal and synaptic signaling, and myelin-dependent axonal connectivity, respectively. Accordingly, NDEs and ODEs are considered here as candidate sources of molecular information related to these two functional compartments. This focus does not exclude potential contributions from astrocyte-, microglia-, or vascular cell–associated EVs, which may provide additional information in future studies.

ODE measurements may provide information related to oligodendrocyte and myelin biology in white matter (14), whereas NDE measurements may provide complementary information regarding neuronal and synaptic processes (11–13, 23) (**Figure 1**). In the present framework, however, NDEs and ODEs should be regarded as operationally defined EV populations enriched using cell-associated surface markers rather than as populations whose brain and cellular origin has been definitively established for every individual vesicle. Our previous studies have used myelin oligodendrocyte glycoprotein (MOG) for enrichment of ODEs and L1 cell adhesion molecule (L1CAM/CD171) for enrichment of NDEs. Although these and other candidate markers can enrich EV populations associated with particular neural cell types, no currently used marker provides absolute brain specificity. Marker expression outside the CNS, heterogeneity of circulating EV populations, and differences in enrichment and detection methods therefore need to be considered when interpreting these populations. Multimarker EV characterization, analysis of associated molecular profiles, orthogonal analytical approaches, and correlation with independent clinical measures may help strengthen their biological interpretation.

Notably, in our recent exploratory study, a marked reduction in circulating ODE levels was observed in an individual who developed chemotherapy-associated cognitive impairment (14). Although this single-patient observation cannot establish an association between ODE abundance and CRCI, it provides a hypothesis for evaluation in larger prospective cohorts. Importantly, changes in the abundance of enriched brain-associated EV populations should not be interpreted directly as loss of tissue integrity, because EV abundance may also be influenced by cellular stress, altered EV release, inflammation, or other biological processes. EV abundance may provide one component of a longitudinal signal, while characterization of EV-associated proteins and other molecular cargo may provide additional information regarding inflammation, cellular stress, repair, degeneration, or other cancer- and treatment-associated processes.

An additional limitation is that circulating brain-associated EV populations cannot currently provide precise anatomical localization. Enrichment using candidate cell-associated markers may provide information regarding putative cellular association but should not be interpreted as regional localization (11, 23, 24). For example, current approaches cannot reliably determine whether circulating NDEs originate from the hippocampus, prefrontal cortex, or other regions implicated in CRCI. Thus, EVs should be considered complementary rather than stand-alone biomarkers: EV measurements may provide molecular and cellular information, whereas neuroimaging provides regional structural and functional information (21–24) (**Figure 1**).

Brain-associated EV populations may also provide molecular information beyond putative cellular association. In addition to intrinsic membrane proteins, EV surfaces can associate with extracellular biomolecules, forming a surface interactome or protein corona that may reflect biological environments encountered by the vesicle (25, 26) (**Figure 1**). Whether such surface-associated molecules reflect the original brain microenvironment or are acquired during transit through biological fluids remains uncertain. Intravesicular cargo can include proteins, lipids, metabolites, and nucleic acids that may reflect aspects of the physiological or pathological state of the cell of origin (27, 28) (**Figure 1**). Combined analysis of EV-associated cellular markers, surface-associated molecules, and molecular cargo could therefore provide complementary information about biological processes underlying CRCI.

Preliminary clinical evidence supports investigation of this concept. In patients with breast cancer receiving anthracycline-based chemotherapy, plasma EV proteomic profiling identified differences between cognitively impaired and unimpaired patients in proteins associated with synaptic plasticity, including dynamin-1, cofilin-1, a disintegrin and metalloproteinase domain-containing protein 10 (ADAM10), and P2X purinoceptor, as well as tight-junction-associated proteins (29). Although these findings were exploratory and derived from plasma EV preparations rather than purified NDEs or ODEs, they provide proof-of-concept that EV molecular profiles may capture treatment-associated biological changes associated with cognitive outcomes.

## Potential implications for oncology practice

Integrating biological measures of brain health into oncology workflows could improve how CRCI is characterized and monitored. If validated prospectively, circulating brain-associated EV populations, including marker-enriched ODEs and NDEs, could provide a minimally invasive approach for repeated assessment of brain-related molecular changes during and after cancer therapy (12, 13). EV-associated molecular profiles may potentially capture biological processes that accompany or precede measurable cognitive changes, although their temporal relationship to cognitive symptoms remains to be established.

The potential clinical value of brain-associated EV measurements lies in their integration with, rather than replacement of, existing CRCI assessments. Neuropsychological testing characterizes the presence, severity, and domains of cognitive dysfunction (15), whereas neuroimaging provides regional information about structural, functional, and connectivity alterations (21). Brain-associated EV measurements could add a complementary molecular dimension related to candidate neuronal and glial processes (11–14). Integrating EV profiles with neuroimaging, neuropsychological performance, and patient-reported symptoms could therefore provide complementary information across molecular, regional brain, and cognitive levels.

In future clinical settings, persistent EV alterations, particularly when accompanied by neuroimaging or cognitive changes, might support closer cognitive follow-up or evaluation of potentially modifiable contributing factors. However, EV findings should not currently guide treatment decisions; any future clinical application will require demonstration of analytical and clinical validity and careful consideration of neurocognitive outcomes alongside oncologic efficacy and survival (7).

Earlier recognition of brain-related changes may also facilitate investigation of supportive interventions aimed at preserving or improving cognitive function. Exercise, cognitive rehabilitation or training, psychosocial interventions, and pharmacological approaches have been investigated in CRCI, although evidence varies across interventions (9, 30). Ultimately, integration of molecular, neuroimaging, and cognitive measures may contribute to a more comprehensive model of survivorship care.

## Future research directions

Realizing the potential of brain-associated EV populations for CRCI monitoring will require prospective longitudinal studies integrating EV measurements with standardized neuropsychological assessments, patient-reported outcomes, and neuroimaging. Ideally, measurements should begin before treatment and continue during therapy and into survivorship, allowing EV trajectories to be related to the onset, persistence, and recovery of cognitive and neuroimaging changes. Such studies will be essential for determining whether EV alterations provide complementary information beyond existing assessment modalities (7, 15, 21).

Methodological standardization will be equally important. Preanalytical factors, including blood collection, processing, storage conditions, and freeze-thaw history, as well as EV isolation and enrichment methods, candidate cell-associated markers, and analytical platforms, can substantially influence EV measurements (24, 27, 28). Harmonized protocols, appropriate controls, and pretreatment baselines will therefore be necessary to establish analytical validity and reproducibility. Particular attention should be given to validating markers used to enrich NDEs and ODEs and determining whether observed changes reflect EV abundance, altered release or clearance, molecular composition, or combinations of these processes (24, 27).

Future studies should also determine which dimensions of EV biology are most informative for CRCI, including cell-associated identity, surface-associated molecules, and intravesicular cargo. Candidate biomarkers of neuroinflammation, oxidative and mitochondrial stress, synaptic dysfunction, and oligodendrocyte/myelin biology should be evaluated in relation to specific cognitive domains and neuroimaging findings. Because circulating EVs cannot currently provide reliable anatomical localization, multimodal studies will be particularly important for determining whether ODE-associated changes correlate with white-matter alterations and whether NDE-associated changes relate to structural or functional alterations in relevant brain networks (21–26). Moreover, comparison of longitudinal EV trajectories between cognitively affected and unaffected patients receiving similar therapies would help distinguish systemic effects of cancer and treatment from EV changes more specifically associated with cognitive outcomes.

Finally, the applicability of this framework across different cancer treatments warrants investigation. Cognitive changes have been associated not only with cytotoxic chemotherapy but also with endocrine therapy, targeted therapies, immunotherapy, and radiation therapy (7, 9). Large prospective cohorts across treatment modalities will be needed to define meaningful longitudinal EV changes and determine their relationship to cognitive outcomes while accounting for potential confounding factors, including age, cancer type and stage, systemic inflammation, fatigue, anemia, sleep disturbance, psychological factors, hormonal changes, medications, and comorbidities. Ultimately, the value of brain-associated EV measurements will depend on demonstrating analytical validity, biological relevance, and incremental value as a complementary molecular component of longitudinal brain health assessment.

## Limitations

The proposed framework is conceptual and hypothesis-generating and has several important limitations. First, changes in EV abundance cannot be interpreted directly as changes in tissue integrity, because EV levels may also reflect altered vesicle release or clearance, cellular stress, inflammation, or other biological processes. Molecular profiling of EV-associated surface and intravesicular components may provide complementary biological information, but the clinical significance of such profiles remains to be established. Second, the cellular and CNS origin of marker-enriched circulating EV populations cannot currently be established with absolute specificity, and candidate enrichment markers may also be expressed outside the CNS. Third, EV measurements are sensitive to pre-analytical and analytical variables, including blood collection and processing, storage conditions, EV enrichment methods, and assay platforms. In addition, cancer itself, anticancer treatment, systemic inflammation, and clinical factors such as age, fatigue, anemia, sleep disturbance, psychological factors, hormonal changes, medications, and comorbidities may influence both EV profiles and cognitive outcomes. Finally, CRCI is heterogeneous, and white- and gray-matter alterations may occur concurrently or in different temporal patterns rather than following a universal sequential progression. Prospective longitudinal studies incorporating pretreatment baselines, appropriate treatment-matched comparisons, cognitive assessments, and neuroimaging will therefore be required to establish the biological and clinical significance of the proposed EV signatures.

Cancer–related cognitive impairment, Liquid biopsy, Biomarkers, Extracellular vesicles, Precision medicine, Brain health.
